# Cancer as trauma: multidimensional determinants of PTSD across the disease course. A narrative integrative review

**DOI:** 10.3389/fpsyg.2025.1719291

**Published:** 2026-01-06

**Authors:** Cittim B. Palomares-Palomares, Eduardo Rios-Garcia, Juan-Manuel Hernandez-Martinez, Jennifer Gómez-Gloria, Luis Llamas-Alonso, Marcela Hernández-Ortega, Luis Lara-Mejía, Georgina Alvarez-Rayón, Narjust Florez, Oscar Arrieta

**Affiliations:** 1 Facultad de Ciencias Administrativas y Sociales, Universidad Autónoma de Baja California (UABC), Ensenada, Mexico; 2 Thoracic Oncology Unit, Instituto Nacional de Cancerología (INCan), Mexico City, Mexico; 3 SECIHTI-Instituto Nacional de Cancerología, Mexico City, Mexico; 4 Centro de Investigación en Ciencias de la Salud (CICSA), Facultad de Ciencias de la Salud, Universidad Anáhuac México, Huixquilucan, Mexico; 5 Centro de Investigación Farmacológica (CIF), Instituto Nacional de Cancerología (INCan), Mexico City, Mexico; 6 Nutrition Research Group, Facultad de Estudios Superiores Iztacala, Universidad Nacional Autónoma de México, Tlalnepantla, Mexico; 7 Lowe Center for Thoracic Oncology, Dana-Farber Cancer Institute, Boston, MA, United States; 8 Harvard Medical School, Boston, MA, United States

**Keywords:** cancer-related PTSD, trauma, quality of life, psychological determinants, socioemotional factors

## Abstract

**Background:**

Post-traumatic stress disorder (PTSD) is a significant psychological response of cancer diagnosis, treatment, and survivorship. This review synthesizes evidence on the psychological, socioemotional, and biomedical determinants of cancer-related PTSD, emphasizing how these factors interact across the disease trajectory.

**Methods:**

We conducted a narrative integrative review of PubMed, PsycINFO, and Scopus. Eligible studies included articles published between 2015 and March 2025 reporting adults with cancer assessed for PTSD using validated instruments across different study designs. Based on findings on prevalence, predictors, and assessment tools a biopsychosocial model was structured.

**Results:**

Twenty-three studies met inclusion criteria, mainly from the United States and China, with breast cancer as the most frequently studied diagnosis. Reported PTSD prevalence ranged widely, from 0 to 72.5%, depending on the instruments and cutoffs used. Psychological determinants included affective comorbidities, fear of recurrence, maladaptive coping, and prior psychiatric history. Socioemotional determinants involved social support, communication quality, demographic variables, and cultural factors. Medical-biological determinants related to treatment aggressiveness, symptom burden, disease stage, and inflammation. Younger age, female sex, and limited social support consistently elevated risk. PTSD was associated with lower quality of life, reduced adherence to treatment, and poorer survivorship outcomes.

**Conclusion:**

Cancer-related PTSD reflects the continuous interaction of psychological, socio-emotional, and medical-biological factors across the cancer journey. These findings underscore the need for culturally sensitive assessment tools and for adapting interventions to the specific demands of each phase of care. Increasing trauma-informed awareness among multidisciplinary teams can enhance early identification of at-risk patients and support patient-centered care.

## Background

The psychological impact experienced by patients with cancer has been recognized and considered a factor that significantly influences the effectiveness of treatments and the recovery of survivors. Among the most severe psychological responses observed is post-traumatic stress disorder (PTSD), which can significantly impair patients’ quality of life and treatment adherence thus hindering the intervention results (Swartzman et al., 2017a; Nipp et al., 2018; Brown et al., 2020; Al Amri et al., 2024).

PTSD is a psychiatric disorder triggered by exposure to a real or perceived threat and is mostly associated with events such as combat, accidents, or natural disasters. However, it is increasingly recognized in medical settings, particularly among individuals facing life-threatening illnesses, such as cancer (Moschopoulou et al., 2018).

Symptoms are characterized by intrusions, avoidance, negative alterations in cognition and mood, and changes in reactivity. Individuals may also experience emotional numbness, detachment, and interpersonal difficulties, all of which can severely disrupt their daily functioning and wellbeing (Lin et al., 2017; Al Jowf et al., 2023). Symptoms must persist for >1 month, cause clinically significant distress observed in functional impairment, and not be attributable to substances or a medical condition (American Psychiatric Association, 2013; Chan et al., 2017; Al Jowf et al., 2023).

From a neurobiological perspective, PTSD reflects a dynamic disturbance across interconnected networks, wherein excessive salience network activity interferes with top-down inhibition from the prefrontal cortex and with contextual encoding in the hippocampus (Hinojosa et al., 2024). In patients with cancer, this architecture becomes uniquely shaped by the nature of oncological stressors. Diagnosis, staging consultations, invasive procedures, chemotherapy, chronic pain, and the pervasive uncertainty surrounding disease progression operate as recurrent threat stimuli that repeatedly activate the amygdala and the hypothalamic–pituitary–adrenal (HPA) axis (Kangas et al., 2002).

**Figure 1:**
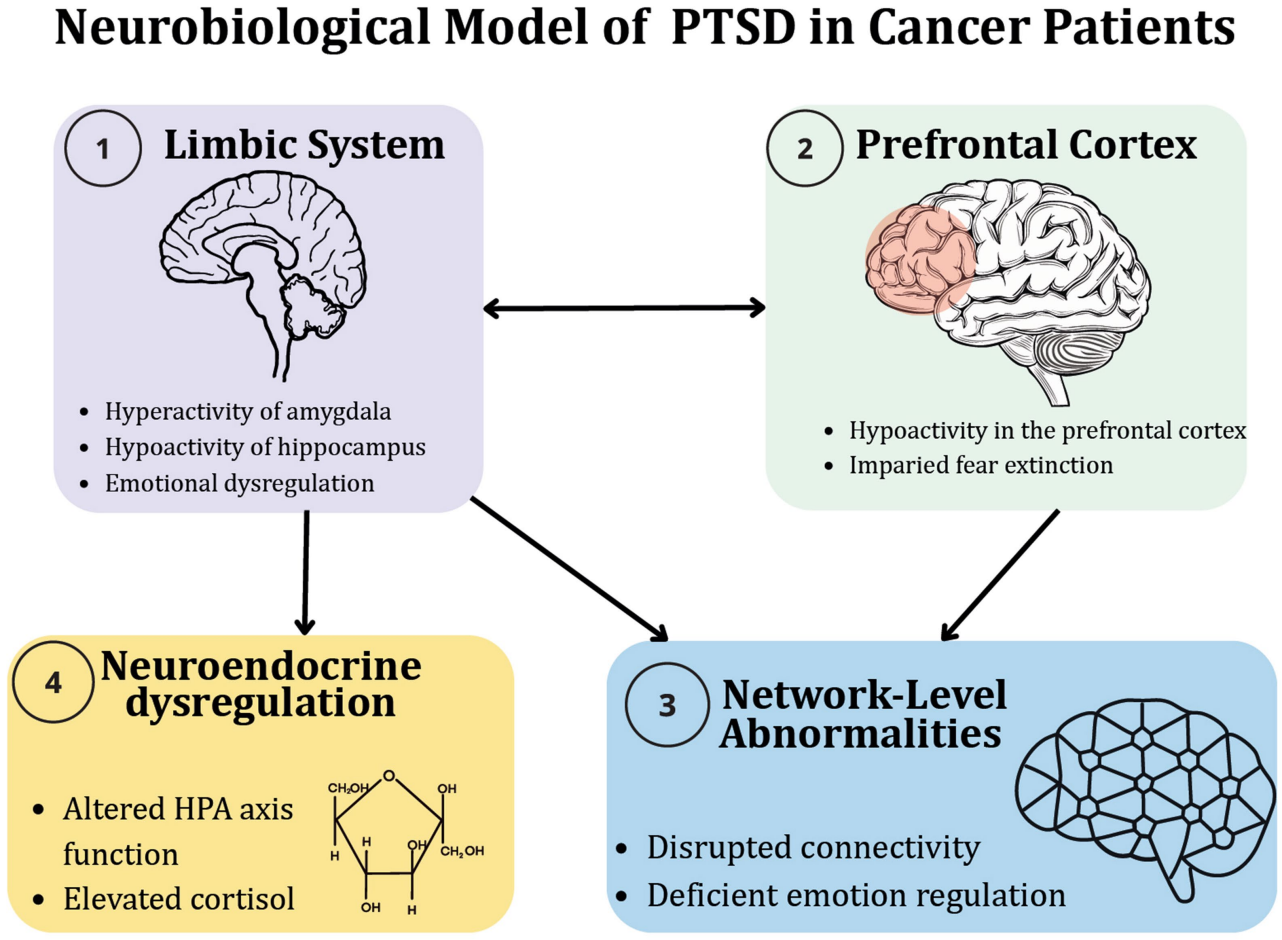
Neurobiological model of PTSD in patients with cancer.

Over time, repeated exposure to hospital environments, medical equipment, and bodily sensations associated with treatment can become conditioned cues that trigger physiological responses similar to those seen in trauma reminders, thereby weakening fear extinction and fostering symptoms of re-experiencing, avoidance, and hypervigilance (Cordova et al., 2017). The model illustrates four core neural and physiological components implicated in PTSD: (1) amygdala hyperactivation, (2) reduced prefrontal regulation (vmPFC, dlPFC), (3) dysregulation of the HPA axis, and (4) hippocampal dysfunction. These alterations sustain heightened threat reactivity, impaired fear extinction, and emotional dysregulation (Figure 1).

In addition to neurofunctional changes, cancer involves a profound psychological and existential changes and, as a result, the course of the disease can be experienced as trauma by patients. In this context, PTSD can emerge as a response to the cumulative and interrelated psychosocial stressors that accompany illness, treatment, and survivorship (Williams et al., 2024; Hinostroza and Mahr, 2025). However, not all patients with cancer develop PTSD. In this regard, associated psychosocial factors have been identified, such as: how the illness is appraised, how social and role environments respond, and how individuals deploy coping and meaning-making in the face of threat (Hinostroza and Mahr, 2025).

Recently, it was reported that psychological distress in cancer survivors is linked to greater intrusive PTSD symptoms, and this association is partly explained by the severity of physical cancer symptoms. It was also found that age and social support moderated outcomes: younger patients with moderate or high support and high symptom burden showed more intrusions, indicating that social support may not always be protective (De Freitas Melo et al., 2024).

The experience of cancer requires studying and understanding it from an integrative perspective, in which neurobiological, medical, and psychosocial elements converge. PTSD in oncology represents a multifactorial and dynamic phenomenon. Rather than arising from a single traumatic event, cancer-related PTSD emerges through the continuous interaction of altered neural circuits, stress-related endocrine and immune dysregulation, and the patient’s subjective appraisal of illness, social context, and coping resources.

While several studies have identified biopsychosocial factors associated with cancer-related PTSD (Schrader and Ross, 2021; Low et al., 2024; Benedict et al., 2025; Nanos et al., 2025), there remains limited clarity regarding how these factors interact across the different stages of the disease trajectory. Therefore, our study sought to conduct a narrative review of recent literature examining the range of biopsychosocial factors implicated in cancer-related trauma and to analyze their interplay throughout the course of the illness.

## Methods

We conducted a systematic search strategy to identify studies evaluating determinants of cancer-related PTSD in adult patients with cancer, following PRISMA 2020 guidelines. We chose this strategy to maximize transparency, completeness, and methodological rigor (Page et al., 2021). However, the review findings were ultimately reported as a narrative review due to the heterogeneous nature of study designs, populations, measures, and outcomes. Full electronic search strategies and exact search dates are detailed in the Supplementary material.

### Search strategy and study selection

We systematically searched three electronic databases, PubMed/MEDLINE, PsycINFO, and Scopus, in March 2025 with a last update on August 2025. Using combinations of controlled vocabulary and free-text terms related to cancer/oncology, PTSD, psychological determinants, socioemotional factors, and quality of life. Search strings were tailored to each database. Reference lists of eligible articles and relevant reviews were hand-searched to identify additional studies.

Records retrieved from the searches were imported into a reference manager, and duplicates were removed. Titles and abstracts were screened to exclude clearly irrelevant reports. Potentially eligible articles were then reviewed in full text to determine inclusion.

### Eligibility criteria

We included peer-reviewed, English-language studies of adult cancer populations (≥18 years) published between 2015 and 2025 that assessed PTSD in patients with cancer with or without caregivers, using validated diagnostic criteria or standardized symptom scales, also employed observational designs; and examined at least one psychological, socioemotional, or medical/biological variable as a correlate or predictor of PTSD.

We excluded single case reports, purely pediatric or adolescent samples and non-empirical publications such as narrative reviews, editorials, comments, and letters without primary data, conference abstracts, and theses or dissertations. When multiple articles used overlapping samples, we prioritized the most comprehensive or methodologically robust report for extraction.

### Data extraction and narrative synthesis

Two reviewers (CBPP and ERG) screened the records identified in the search by examining titles and abstracts to determine potential eligibility. Full-text versions were then obtained for all articles that appeared to meet the criteria. Two authors (JMHM and JGG) independently assessed each full-text article against the inclusion criteria through detailed reading. Any disagreements were resolved through discussion with a third author (LLA).

From each included study, we extracted: study design; country and setting; sample size and cancer type; timing of PTSD assessment relative to the disease trajectory; instruments and cut-offs used (e.g., PCL-C, PCL-5, IES-R, DSM-based criteria); prevalence estimates; and variables evaluated as correlates or predictors of PTSD. We focused on multivariable analyses, recording effect sizes and their confidence intervals when reported.

To structure the synthesis, extracted determinants were grouped *a priori* into three domains, consistent with a biopsychosocial perspective: psychological, socioemotional, and medical/biological. Within each domain we made further classifications according to the findings, we distinguished between variables examined only in unadjusted analyses and those retained as independent predictors in multivariable models.

### Quality assessment

Methodological quality and risk of bias of the included observational studies were appraised using the Observational Study Quality Evaluation (OSQE) tool, applying the version appropriate to each study design (Drukker et al., 2021). For each included study, we derived a total OSQE score and examined the distribution of scores across the corpus to contextualize the strength and consistency of the evidence. Quality ratings were used descriptively to qualify the narrative synthesis rather than as hard thresholds for inclusion or exclusion.

### Biopsychosocial modeling

Guided by Engel’s biopsychosocial framework (Engel, 1977; Papadimitriou, 2017), we synthesized the evidence identified in this review to build a conceptual model of cancer-related PTSD that integrates biological vulnerability, psychological processes, and social-contextual factors. We first mapped risk and protective factors within each domain, then iteratively organized their relationships with PTSD symptoms, trajectories, and clinical outcomes. This model was refined through team discussion and constant comparison with the included studies to highlight plausible mechanisms and potential leverage points for assessment and intervention.

## Results

**Figure 2:**
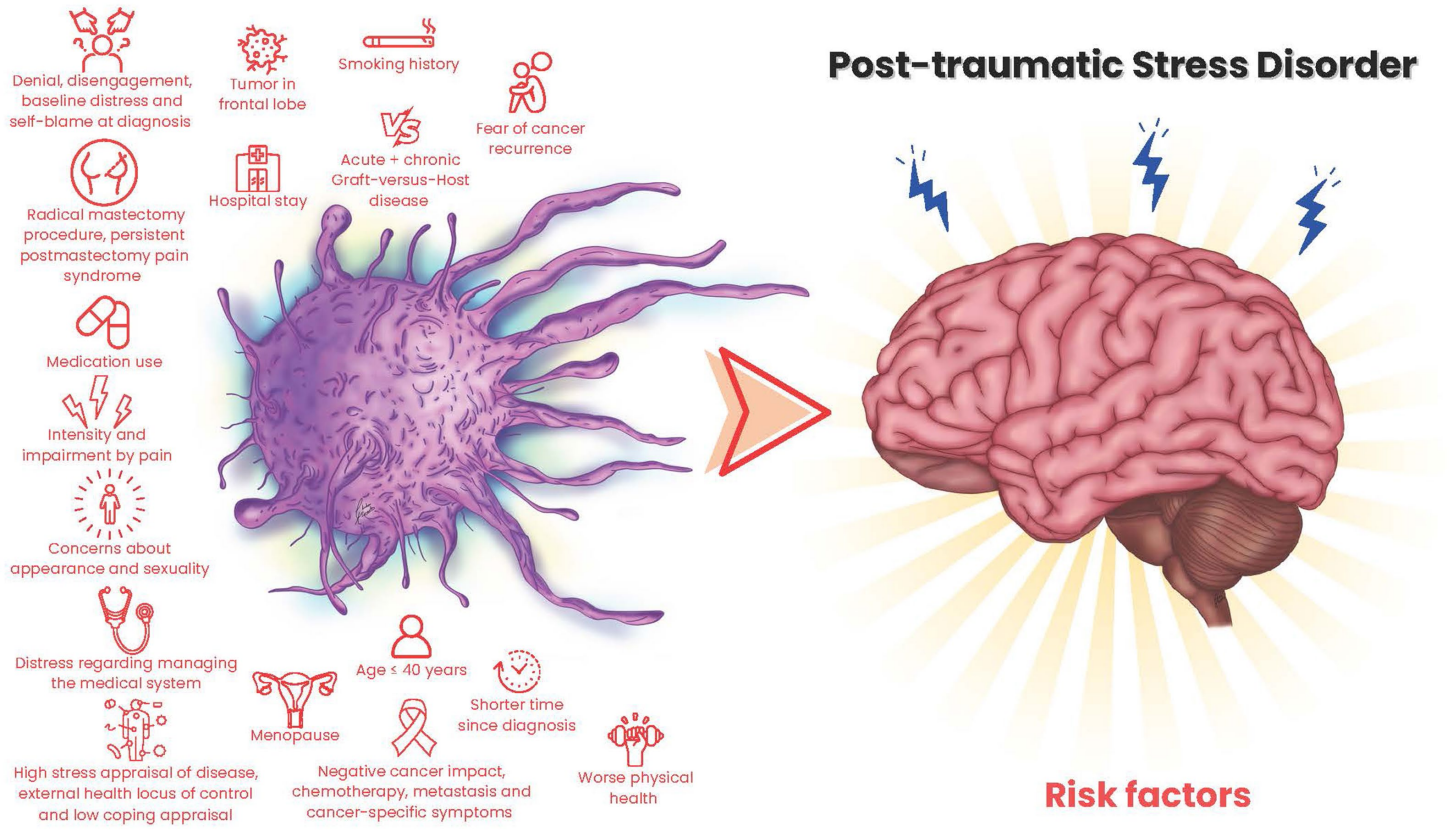
Cancer-related risk factors for PTSD.

As a result of our comprehensive search, we identified 1,262 records. Of these, 116 were assessed for eligibility and 19 met the inclusion criteria. Screening the reference lists of the included articles yielded 4 additional studies, which were also included (Supplementary Figure S1). Across the 23 studies, the main predictive variables of PTSD in cancer patients were classified in psychological, socio-emotional, and medical-biological dimensions. Key variables included maladaptive coping mechanisms, secondary trauma in caregivers, comorbidities, and disease stage. We propose a biopsychological model of PTSD that synthesizes risk and protective factors, and suggests viable interventions (Table 1 and Figure 2).

**Table 1:** Summary of the reviewed studies examining cancer-related PTSD.

Author	Design of the study	Sample size (% of females) Age mean (SD)	Type of cancer	Statistical analysis	PTSD assessment	PTSD frequency	Risk factors	Protective factors
Hahn et al. (2015) USA	Cross-sectional	162 (85% female) 51 (16.0)	Mixed: breast (60%), hematologic, pediatric	Multivariate linear, logistic regression	PTSD checklist-Civilian (PCL-C; cut-offs: 30, 38, 44)	29% (PCL-C ≥ 30), 13% (≥38), and 7% (≥44); mean score = 27 (*SD* = 9.0)	Negative cancer impact (*β* = 4.17, *p* < 0.001), depression (*β* = 4.59, *p* = 0.003), low social support (*β* = −2.42, *p* = 0.06), low mental health (*β* = −0.21, *p* = 0.001), lower income (*β* = 2.75, *p* = 0.03), and being married (*β* = 2.22, *p* = 0.04)	High social support (*β* = −2.44, *p* = 0.04), quality of life (*β* = −0.21, *p* = 0.001)
Alkan et al. (2016) Turkey	Multicenter cross-sectional	614 (100% female) 54.4 (10.1)	Breast cancer (remission)	Univariate, multivariate logistic regression	PCL-C (cut-off ≥24)	72.5%; mean score = 32.4 (*SD* = 11.1)	Persistent postmastectomy pain syndrome (*OR* = 3.02, *CI* = 2.01–4.55, *p* < 0.001) and being married (*OR* = 1.57, *CI* = 1.00–2.41, *p* = 0.03)	None
El-Jawahri et al. (2016) USA	Prospective longitudinal	90 (41.1% female) 58.1 (14.4)	Hematologic malignancies (HCT patients)	Multivariable linear regression	PTSD Checklist for DSM-5 (PCL-5)	28.4% at 6 months post-HCT	Quality of life during hospitalization (*β* = −0.40, *p* < 0.0001), depression (*β* = +1.26, *p* < 0.0001), and anxiety (*β* = +1.33, *p* = 0.002)	Being married (*β* = −5.70, *p* = 0.03)
Richardson et al. (2016) New Zealand	Prospective longitudinal	65 (28% female) 61.8 (not reported)	Head and neck cancer (± advanced skin)	Hierarchical multiple regression	PTSD scale-self report for DSM-5 (PSS-SR5; cut-off >13)	19% at 6 months post-diagnosis	Denial (*β* = 0.23, *p* = 0.027), behavioral disengagement (*β* = 0.25, *p* = 0.013), baseline distress (*β* = 0.25, *p* = 0.021), and self-blame at diagnosis (*β* = 0.30, *p* = 0.005)	None
Esser et al. (2017) Germany	Prospective longitudinal	239 (38% female at baseline) 50.4 (13.6)	Hematologic malignancies (allogeneic HSCT patients)	Multilevel modeling, multiple regression	PCL-C (cut-off ≥50)	3–6% (sum score method), 8–15% (symptom-based method), and 52% had significant intrusion	Being a woman (*γ* = 3.81, *p* < 0.01), pain intensity (*γ* = 0.63, *p* = 0.02), impairment by pain (*γ* = 2.89, *p* < 0.01), longer hospital stay (*γ* = 0.10, *p* = 0.03), and acute + chronic graft-versus-host disease (*γ* = 3.39, *p* = 0.04)	None
Kuba et al. (2017) Germany	Multicenter prospective longitudinal	239 (38% female) 50.4 (12.0)	Hematologic malignancies (allogeneic HSCT patients)	Multilevel modeling (random intercept models), regression analyses	PTSD Checklist for DSM-IV	Not specified	Concerns about appearance and sexuality before HSCT versus 3 (*β* = 0.40, CI = 0.25–0.54, *p* < 0.001, *R^2^* = 0.17) and 12 months later (*B* = 0.18, *p* = 0.03, *CI* = 0.01–0.35, *R^2^* = 0.29). Distress regarding managing the medical system before HSCT 12 months after HSCT (*β* = 0.28, *p* = 0.01, *CI* = 0.09–0.47, *R^2^* = 0.29)	None
Lin et al. (2017) Taiwan	Cross-sectional	347 (77% female) 56.3 (12.0)	Breast, gynecological, colorectal cancer	Multivariate logistic regression	Chinese Davidson Trauma Scale (C-DTS)	~30% (C-DTS > mean); mean score = 22.85 (*SD* = 24.12)	Suicidal intention (*OR* = 2.29, *CI* = 1.86–2.82, *p* < 0.001), chemotherapy (*OR* = 2.13, *CI* = 1.18–3.84, *p* = 0.012), metastasis (*OR* = 2.07, *CI* = 1.29–3.34, *p* = 0.003), high education (*OR* = 1.75, *CI* = 1.10–2.78, *p* = 0.018), and cancer-specific symptoms (*OR* = 1.21, *CI* = 1.15–1.27, *p* < 0.001), e.g., fever, dry mouth, bowel changes, pain, vomiting, insomnia	None
Swartzman et al. (2017a,b) Scotland	Cross-sectional survey	205 (39.5% female) 71.0 (8.2)	Colorectal cancer	Multiple linear regression, mediation analysis (PROCESS)	PCL-C (modified for cancer context; cut-off ≥50)	4.4%; mean score = 24.0 (*SD* = 10.2)	Family conversational constraints (*β* = 0.54, *p* < 0.001) and lower age (*β* = −0.20, *p* = 0.001)	Family identification (*β* = −0.14, *p* = 0.03) and social support (*β* = −0.24, *p* < 0.01)
Chan et al. (2017) Malaysia	Prospective cohort (4 years)	469 (63% female) 56.2 (13.0)	Mixed	Chi-square, ANOVA, logistic regression	Structured Clinical Interview for the Diagnostic (SCID, DSM-IV-TR)	21.7% (6 months), 6.1% (4 years), and 34.1% had persistent or worsening PTSD over time	None	At 6-month follow up Breast cancer (*OR* = 0.272 *CI* = 0.089–0.834 *p* = 0.023)
Allen et al. (2018) USA	Cross-sectional, SJLIFE cohort	2,969 (49.1% female) 32.5 (8.5); 24.1 years since diagnosis	Childhood cancer (multiple diagnoses, survivors >10 years)	Multivariable linear regression with Bayesian model averaging	PCL-C (cut-off ≥44)	11.8%; mean score = 27.7 (*SD* = 12.4)	Female Estimate = 1.64, SE = 0.28, *p* < 0.0001, less than college education Estimate = 0.90, SE = 0.30, *p* = 0.002, high perceived stress estimate = 0.76, SE = 0.05, *p* < 0.0001, high psychological distress estimate = 0.67, SE = 0.02, *p* < 0.001, high cancer-related anxiety estimate = 0.10, SE = 0.03, *p* = 0.001	None
Ni et al. (2018) China	Prospective longitudinal	93 (28% female) 62 (10)	Lung cancer	Linear regression, subgroup analysis	PCL-C (cut-off ≥50)	0% full PTSD at baseline or 6 months; 64% mild, and 8% moderate at 6 months; PTSD scores significantly worsened over time	Younger age (*β* = 0.34, *p* = 0.003), low smoking history at diagnosis (*β* = 0.29, *p* = 0.012), less sedentary time at diagnosis (*β* = 0.39, *p* = 0.005), worse health-related quality of life (*β* = 0.33, *p* = 0.004), fatigue (*β* = 0.37, *p* = 0.001), nausea/vomiting (*β* = 0.39, *p* = 0.001), pain (*β* = 0.36, *p* = 0.002), appetite loss (*β* = 0.55, *p* = 0.005), constipation (*β* = −7.50, *p* = 0.002)	None
Jiang and Wang (2019) China	Prospective cohort (5-year follow-up)	219 (38% female) 41.5 (10.9)	Low-grade glioma	Logistic regression, Cox proportional hazards	Impact of Event Scale-Revised (IES-R; cut-off >33)	16% at 3 months postop; PTSD predicted lower 5-year survival (HR = 2.98)	Age ≤ 40 years (*β* = 2.23, *p* = 0.04), tumor in frontal lobe (*β* = 2.57, *p* = 0.04), anxiety (*β* = 3.26, *p* < 0.001), depression (*β* = 3.46, *p* < 0.001)	None
Zhou et al. (2019) China	Cross-sectional, LPA-based	191 (67% female) 51.6 (10.7)	Mixed: gastric, liver, breast, thyroid, uterine, etc.	Latent profile analysis, multinomial logistic regression	PCL-5	15.3% no symptoms, 44.2% hyperarousal group, and 40.5% severe group	Fear of cancer recurrence (*OR* = 1.32, *CI* = 1.18–1.47, *p* < 0.001), being a woman (*OR* = 0.37, *CI* = 0.16–0.87, *p* = 0.02), household income (*OR* = 1.65, *CI* = 0.96–2.84, *p* < 0.08), and shorter time since diagnosis (OR = 0.98. *CI* = 0.95–1.00, *p* < 0.08)	None
Acquati et al. (2020)	Cross-sectional, baseline data from intervention study	96 (100% female) 54.5 (7.91)	Breast cancer	Hierarchical multiple linear regression	PSS-SR (cut-off = 13)	54.2%	Final model: social constraints in communication (*β* = 0.40, *p* = 0.001), and worse physical health (*β* = −0.22, *p* = 0.05)	Social support (*β* = −0.14, *p* = 0.03) 1 in final model *β* = −0.10, *p* = 0.12.
MoshirPanahi et al. (2020) Iran	Cross-sectional	300 (55.7% female) 53.0 (27.6)	Breast, leukemia, colorectal cancer	Structural equation modeling	PCL-5	Mean score = 32.4 (*SD* = 13.0); frequency not explicitly	Negative attentional bias (*β* = 0.05, *p* < 0.001), negative cognitive processing (*β* = 0.81, *p* < 0.001), indirect effects through maladaptive cognitive (denial, regret) pathways	None
Simunovic and Ljubotina (2020) Croatia	Cross-sectional, mixed-method	97 (100% female) 50.0 (10.3)	Breast cancer	Hierarchical multiple regression	PCL-C (cut-off = 50)	21.6% full PTSD and 32% with symptoms; Mean score = 38.8 (*SD* = 14.1)	Stress appraisal of disease (*β* = 0.45, *p* = 0.001), external health locus of control (*β* = 0.17, *p* = 0.05), coping appraisal (*β* = −2.82, *p* = 0.048), and radical mastectomy procedure (*β* = 8.78, *p* = 0.003)	Coping appraisal (*β* = −0.17, *p* = 0.05)
Chu et al. (2021) USA	Cross-sectional, baseline of RCT	136 (100% female) 57.8 (9.2)	Breast cancer	Hierarchical linear regression	PSS-SR	59.6%	Social constraints (*β* = 0.45, *p* = 0.001), low income (*β* = −0.20, *p* = 0.05), tangible support not effective or worsened PTSD (*β* = 0.23, *p* = 0.04)	Support of positive interaction buffered PTSD symptoms (*β* = −0.35, *p* = 0.04)
De Padova et al. (2021) Italy	Cross-sectional, dyadic study	212 dyads (58.5% female survivors) Survivors: 59.3 (14.8)	Mixed cancers (breast, testicle, GI, GU, etc.)	Multiple regression, Actor–partner interdependence models	Impact of Event Scale (IES)	Survivors: 20% possible PTSD and 11% probable PTSD; Caregivers: 35.5% possible PTSD and 15.6% probable PTSD	Anxious coping (*β* = 0.55, *CI* = 0.25–0.86, *p* = 0.001), prior psychopathology (*β* = −1.36, *CI* = −2.64−−0.08, *p* = 0.038), depression (*β* = 0.62, *CI* = 0.502–0.729, *p* = 0.001), and being a woman (*β* = 0.97, *CI* = 0.21–1.73, *p* = 0.012)	None
Jung et al. (2021) USA	Cross-sectional, population-based	376 (27.7% female) 72.2 (9.2)	Non-muscle-invasive bladder cancer	Hierarchical multiple linear regression	PCL-5 (cut-off ≥31)	5.3% (observed), 6.9% (adjusted for nonresponse); 28.7% had ≥1 PTSD symptom cluster	Younger age (*β* = −0.20, *p* < 0.01), not cured (*β* = 6.60, *p* < 0.001), uncertain disease status (*β* = 4.40, *p* < 0.001), more comorbidities (*β* = 1.00, *p* < 0.001), and cognitive concerns (*β* = 0.50, *p* < 0.001)	Social support (*β* = −0.10, *p* < 0.05)
Marino et al. (2022) France	Cross-sectional, postal survey post-lockdown	1,097 (63.2% female) 22–92 (47.7% aged 51–70)	Mixed: breast, hematologic, digestive, urologic	Multivariate logistic regression	IES-R (cut-off ≥33)	14.7, and 30.5% had clinical anxiety	Fear of coming to hospital due to COVID-19 (*OR* = 3.49, *CI* = 2.11–5.79, *p* = 0.001), being a woman (*OR* = 1.97, *CI* = 1.18–3.29, *p* = 0.009), living alone (*OR* = 1.63, *CI* = 1.01–2.63, *p* = 0.045), negative lockdown experience (*OR* = 0.98, *CI* = 0.97–0.99, *p* = 0.001), and previous difficult life events (*OR* = 2.11, *CI* = 1.45–3.07, *p* = 0.001)	Positive lockdown experience (*OR* = 0.98, *CI* = 0.97–0.99, *p* < 0.001)
Al Amri et al. (2024) Oman	Cross-sectional, Prospective, multicentered, observational study	343 (100% female)	Breast cancer	Logistic regression	PCL-5	27.4%	Shorter time since diagnosis (*OR* = 0.98, *CI* = 0.97–0.99, *p* = 0.017), no psychiatric referral (*OR* = 0.46, *CI* = 0.23–0.96, *p* = 0.04), and insufficient social support (*OR* = 0.96, *CI* = 0.94–0.98, *p* < 0.001).	Enhanced social support (*OR* = 0.95, *CI* = 0.93–0.97, *p* < 0.001)
Xiao et al. (2025) China	Cross-sectional	674 (100% females) 48.50 (10)	Breast cancer	Multiple linear regression analysis	PCL-5	13.9%	Ethnicity (*β* = 0.06, *p* = 0.045), negative coping (*β* = 0.18, *p* < 0.001), and expression inhibition (*β* = 0.11, *p* = 0.004)	Internal locus of control (*β* = −0.14, *p* < 0.001), subjective support (*β* = −0.13, *p* = 0.002), and cognitive reappraisal (*β* = −0.16, *p* < 0.001)
Zhou et al. (2025) China	Cross-sectional, prediction model (TOUS-based)	1,135 (100% female) Median = 54 (IQR 47–62)	Breast cancer	Multivariate logistic regression, nomogram	PCL-C (cut-off ≥50)	24.1% (training); 35.1% (validation cohort)	Menopause (*OR* = 0.52, *CI* = 0.33–0.80, *p* < 0.001), high blood cholesterol (*OR* = 1.46, *CI* = 1.24–1.73, *p* < 0.001), fear of cancer progression (*OR* = 1.07, *CI* = 1.05–1.10, *p* < 0.001), psychological distress (*OR* = 6.81, *CI* = 3.67–13.53 p < 0.001), depression (*OR* = 1.77, *CI* = 1.10–1.24, *p* < 0.001), and current smoking (*OR* = −1.56, *CI* = 0.12–0.37, *p* < 0.001).	Social support (*OR* = 0.88, *CI* = 0.84–0.91, *p* < 0.001), never/former smoking (*OR* = 0.36, *CI* = 0.18–0.69, *p* < 0.001), and premenopause (*OR* = 0.52, *CI* = 0.33–0.80, *p* < 0.001)

PCL-C, PTSD Checklist–Civilian Version; PCL-5, PTSD Checklist for DSM-5; PSS-SR: PTSD Symptom Scale–Self Report; IES-R, Impact of Event Scale–Revised; IES, Impact of Event Scale; C-DTS, Chinese Davidson Trauma Scale; DSM-IV/5, Diagnostic and Statistical Manual of Mental Disorders, Fourth/Fifth Edition; HSCT, Hematopoietic Stem Cell Transplantation; HCT, Hematopoietic Cell Transplantation; GI, Gastrointestinal; GU, Genitourinary; RCT, Randomized Controlled Trial; SJLIFE, St. Jude Lifetime Cohort; APIM, Actor–Partner Interdependence Model; TOUS, theory of unpleasant symptoms; SD, standard deviation; OR, odds ratio; CI, confidence interval; *β*, standardized regression coefficient; *γ*, multilevel model coefficient; HR, hazard ratio; *R*^2^, coefficient of determination.

Statistical significance was considered at *p* < 0.05 unless otherwise stated. PTSD frequency and severity may differ based on the assessment tool and cut-off used.

Studies were conducted across countries, mainly in the USA (26.1%) and China (21.7%). Only seven studies were longitudinal (30.4%), while others were cross-sectional. The studies’ generalizability is limited due to varied patient populations. Breast cancer was the most studied type (52%). PTSD assessment tools included the PTSD Checklist-Civilian version [PCL-C; (Weathers et al., 1993)], PTSD Checklist for DSM-5 [PCL-5; (Blevins et al., 2015)], Impact of Event Scale-Revised [IES-R; (Weiss)], and DSM-IV criteria. PTSD rates ranged from 0% (Ni et al., 2018) to 72.5% (Alkan et al., 2016), varying by assessment tool and criteria. With stricter detection criteria, rates ranged from 0% (Ni et al., 2018) to 40.5% (Zhou et al., 2019), and varied over time after surgery or during follow-up periods.

### Psychological determinants

Elevated psychological distress is linked with increased PTSD symptoms in cancer patients. Depressive (El-Jawahri et al., 2016; De Padova et al., 2021; Zhou et al., 2025) and anxiety symptoms (Jiang and Wang, 2019; De Padova et al., 2021) often co-occur with PTSD. Fear of recurrence or progression is a distinct feature of distress in oncology, identified as a strong PTSD predictor (Hahn et al., 2015; Zhou et al., 2019; Marino et al., 2022; Zhou et al., 2025). This aligns with limited evidence on other serious conditions like cardiac events (Fait et al., 2018; Cui et al., 2022), strokes (Noble et al., 2011; Guan et al., 2024), or schizophrenia (White and Gumley, 2009).

Patients with cancer experiencing distress related to body image, sexuality, physical limitations, or healthcare system navigation report higher PTSD symptom burden (Kuba et al., 2017). These concerns intensify feelings of vulnerability and loss of control, leading to trauma-related avoidance (Simunovic and Ljubotina, 2020). Healthcare system navigation difficulties, established as a barrier to PTSD treatment in war veterans (Rozek et al., 2023), appear to extend to cancer patients with PTSD.

Cognitive processing styles significantly influence PTSD symptoms. Negative cognitive appraisals, including self-blame and catastrophic thinking, are major risk factors for PTSD severity (MoshirPanahi et al., 2020). Cognitive concerns in attention and executive functioning can undermine emotional regulation (Jung et al., 2021). Patients using maladaptive coping mechanisms often experience persistent PTSD symptoms (Richardson et al., 2016), which interfere with processing cancer-related trauma and psychological recovery.

Beliefs about personal control over health outcomes play a critical role in psychological resilience and vulnerability. In cancer patients, an external locus of control has been associated with increased PTSD severity (Simunovic and Ljubotina, 2020); conversely, an internal locus of control has been linked to reduced PTSD risk (Xiao et al., 2025). Similar results have been shown in several previous studies on natural disasters (Song et al., 2021; Tian et al., 2022; Guzel et al., 2024) and survivors of severe acute respiratory syndrome (Mak et al., 2010).

History of psychiatric illness, anxiety and/or depressive disorders, markedly elevates the risk of developing PTSD following a cancer diagnosis (De Padova et al., 2021). These patients might not perceive cancer as a unique traumatic event but rather as an aggregate or compounding stressor. Likewise, suicidal ideation has been documented in patients that exhibit a significant PTSD symptom burden, especially among those with a bad prognosis, unaddressed pain, or social isolation (Lin et al., 2017). This points out the significance of proactive screening, psychiatric referrals, and integrated mental health care, especially for patients with psychological risk factors (Al Amri et al., 2024).

### Socioemotional determinants

Recovering from trauma depends on being able to talk to each other and feel connected. In oncology settings, emotional inhibition has been linked to aggravated PTSD symptoms (Xiao et al., 2025). Patients who are unable to express their fears and concerns endure increased psychological isolation (Acquati et al., 2020). Perceived social support, particularly from family, serves as a protective factor against the development of PTSD in cancer patients. Low family identification and weakened supportive networks correlate with heightened PTSD symptomatology (Hahn et al., 2015; Swartzman et al., 2017b; Jung et al., 2021), whereas subjective support functions as a protective factor (Al Amri et al., 2024; Zhou et al., 2025). The quality of interpersonal communication affects the risk of PTSD; this is clear in non-cancer patients who struggle with emotional expression and have higher levels of PTSD (Blais et al., 2021; Liu et al., 2021). These relational dynamics exacerbate psychological vulnerabilities, further intensified by compromised emotional communication, as evidenced in breast cancer patients (Williamson et al., 2021; Kallay et al., 2022; Zhao et al., 2022; Futterman et al., 2023; Malgaroli et al., 2023).

Sociodemographic characteristics influence the structural and relational contexts in which cancer-related trauma occurs. These factors influence an individual’s capacity to manage stressors and their accessibility to psychosocial resources and recovery opportunities. Consistently, female gender and younger age have been associated with an increased risk of PTSD among patients with cancer (Esser et al., 2017; Allen et al., 2018; Ni et al., 2018; Jiang and Wang, 2019; Zhou et al., 2019; Jung et al., 2021; Marino et al., 2022). These patterns may signify heightened emotional sensitivity, modified social roles and expectations, and increased disruption to developmental or reproductive life goals (Zebrack, 2011; Abbey et al., 2015; Kaster et al., 2019; Brown et al., 2020; Vazquez et al., 2020; Chen et al., 2023).

The effect of marital status is even more complex. El-Jawahri suggested in a sex-mixed sample that marriage or a committed relationship provides emotional and instrumental support that reduces the risk of PTSD in patients (El-Jawahri et al., 2016), On the other hand, Alkan found that the partners of women with breast cancer may make their mental health worse (Alkan et al., 2016). This could be linked to stress from caregiving, gendered expectations, or unequal emotional exchange.

Findings related to socioeconomic status are similarly inconclusive. The severity of PTSD symptoms has been linked to both low and high income (Hahn et al., 2015; Zhou et al., 2019), as well as high and low education (Lin et al., 2017; Allen et al., 2018), have been linked to PTSD symptom severity. These contradictions suggest that occupational demands, financial stress, access to psychosocial services, and health literacy may function as mediating variables in the association between socioeconomic status and trauma-related outcomes. Finally, being a minority or of a certain ethnicity raises the risk of PTSD. People who are not part of a minority group often say they have fewer PTSD symptoms. This may be because they get more support from the system and their cultural values fit better with healthcare systems (Xiao et al., 2025). People from minority backgrounds may, however, feel more stressed because of differences in healthcare, cultural stigma, or a history of discrimination.

Cultural and linguistic factors affect how cancer-related PTSD shows up, but studies from both the U.S. and China have found that the same things can cause PTSD: being younger, having a lot of symptoms, being already stressed, not getting enough help, and having financial problems. U.S. studies have notably highlighted financial and occupational stressors as predictors of PTSD within Chinese populations. Measurement heterogeneity limits interpretation: studies utilized Western-developed scales (PCL-C with cutoffs 24–50, PCL-5 cutoff 31, IES-R cutoff 33) and DSM criteria to define “probable PTSD, “leading to increased prevalence with lenient thresholds and conservative estimates with rigorous methodologies. The rates were elevated in high-risk scenarios, yet diminished among long-term survivors. PTSD in oncology is comprehended via Western paradigms; cross-cultural research indicates variability in symptom prominence, and mere translation fails to guarantee conceptual equivalence, thus necessitating DSM-5’s Cultural Concepts and the development of localized assessments (Hinton and Lewis-Fernandez, 2011; Kohrt et al., 2014; Heim et al., 2022).

### Secondary or shared trauma in oncology caregivers

The psychological effects of cancer-related PTSD affect both patients and their caregivers. De Padova et al. (2021) discovered that 20% of long-term survivors and 35.5% of caregivers exhibited PTSD symptoms, 11% of patients and 15.6% of caregivers satisfied the criteria for probable PTSD. Caregivers may undergo secondary trauma upon witnessing a loved one’s suffering, invasive procedures, or threat cues. In samples free from disease, caregivers demonstrated clinically significant PTSD. De Padova et al. (2021) reported substantial caregiver burden, with many individuals fulfilling probable PTSD criteria. A dyadic study in head-and-neck cancer examined the interrelationship between partner–patient distress and caregiver PTSD (Moschopoulou et al., 2018). Research demonstrates that cancer can trigger PTSD in caregivers and indicates that factors such as age, sex, and income may affect vulnerability and the need for intervention (Benyo et al., 2023; Low et al., 2024; Nanos et al., 2025). These data advocate for the continuous assessment of caregiver distress and the integration of caregiver outcomes within psycho-oncology care pathways and clinical trials.

### Medical and biological determinants

Aggressive cancer treatments are linked to a higher risk of PTSD. Patients undergoing chemotherapy (Lin et al., 2017; Acquati et al., 2020), radical surgeries like mastectomy (Simunovic and Ljubotina, 2020), or experiencing postoperative complications (Alkan et al., 2016) show elevated PTSD symptoms. Cancer symptoms such as fatigue (Ni et al., 2018), dry mouth, bowel changes (Lin et al., 2017), appetite loss (Ni et al., 2018), and pain (Esser et al., 2017) increase PTSD risk. Patients with metastatic disease (Lin et al., 2017), active disease (Jung et al., 2021), or frontal lobe tumors (Jiang and Wang, 2019) may experience emotional dysregulation, intensifying trauma responses.

A big risk factor is not knowing how the disease will progress. Patients with unclear prognoses or not enough medical information are more likely to be distressed, which makes them more hypervigilant and emotionally unstable (Kuba et al., 2017; Jung et al., 2021). Longer hospital stays have been linked to worse PTSD symptoms because of longer exposure to medical settings, changes in routine, and less freedom (Esser et al., 2017).

Certain patient characteristics, particularly premenopausal status, have been linked to a diminished risk of developing PTSD in specific cancer cohorts, indicating that biological and hormonal factors may affect resilience pathways (Zhou et al., 2025).

The time since diagnosis affects how bad PTSD is, with a shorter time leading to more hyperarousal and distress (Zhou et al., 2019; Al Amri et al., 2024). While symptoms may lessen as patients adjust, PTSD can persist or intensify, especially in instances of recurrent illness or inadequate support. These findings align with studies demonstrating that treatment and disease-specific stressors increase psychological vulnerability in cancer populations (Rogiers et al., 2020; Malgaroli et al., 2023; Mitchell et al., 2023; Al Amri et al., 2024; Sabah et al., 2025). The physical manifestations of cancer serve as both direct sources of trauma and aggravators of emotional distress.

A review indicated that more than one-third of breast cancer patients initially diagnosed with PTSD exhibited enduring or exacerbating symptoms 4 years later (Brown et al., 2020). Longitudinal studies indicate that breast cancer patients may initially exhibit trauma-related symptoms, yet they are less likely to fulfill complete diagnostic criteria at 6 months in comparison to other cancers (Chan et al., 2017).

### Quality of life

Health-related quality of life (HRQoL) exists in all three dimensions, evident in cancer patients and their caregivers due to the physical and emotional burdens of caregiving. Studies indicate that patients suffering significant psychological distress from a cancer diagnosis report diminished quality of life and heightened PTSD symptoms (Hahn et al., 2015). Low initial HRQoL and compromised physical health forecast the onset and continuation of PTSD (El-Jawahri et al., 2016; Jiang and Wang, 2019; Acquati et al., 2020). Lifestyle and functional factors correlate with the severity of PTSD. Patients exhibiting reduced sedentarism, limited smoking at diagnosis, or diminished appetite demonstrated an elevated risk of PTSD (Ni et al., 2018), potentially indicating increased physiological sensitivity or systemic distress responses. These associations suggest a complicated relationship between physical condition, behavioral reactions, and trauma-related symptoms. Evidence indicates that diminished HRQoL across various dimensions forecasts increased PTSD severity. Fatigue, sleep problems, and pain are common symptoms in oncology that can make emotional distress worse. This can make the cycle of physical decline and trauma continue (Schelling et al., 1998; Danielsson et al., 2018; Rogiers et al., 2020; Hamrick et al., 2023; Mitchell et al., 2023; Miller et al., 2024).

### Biopsychosocial model of cancer-related PTSD

**Figure 3:**
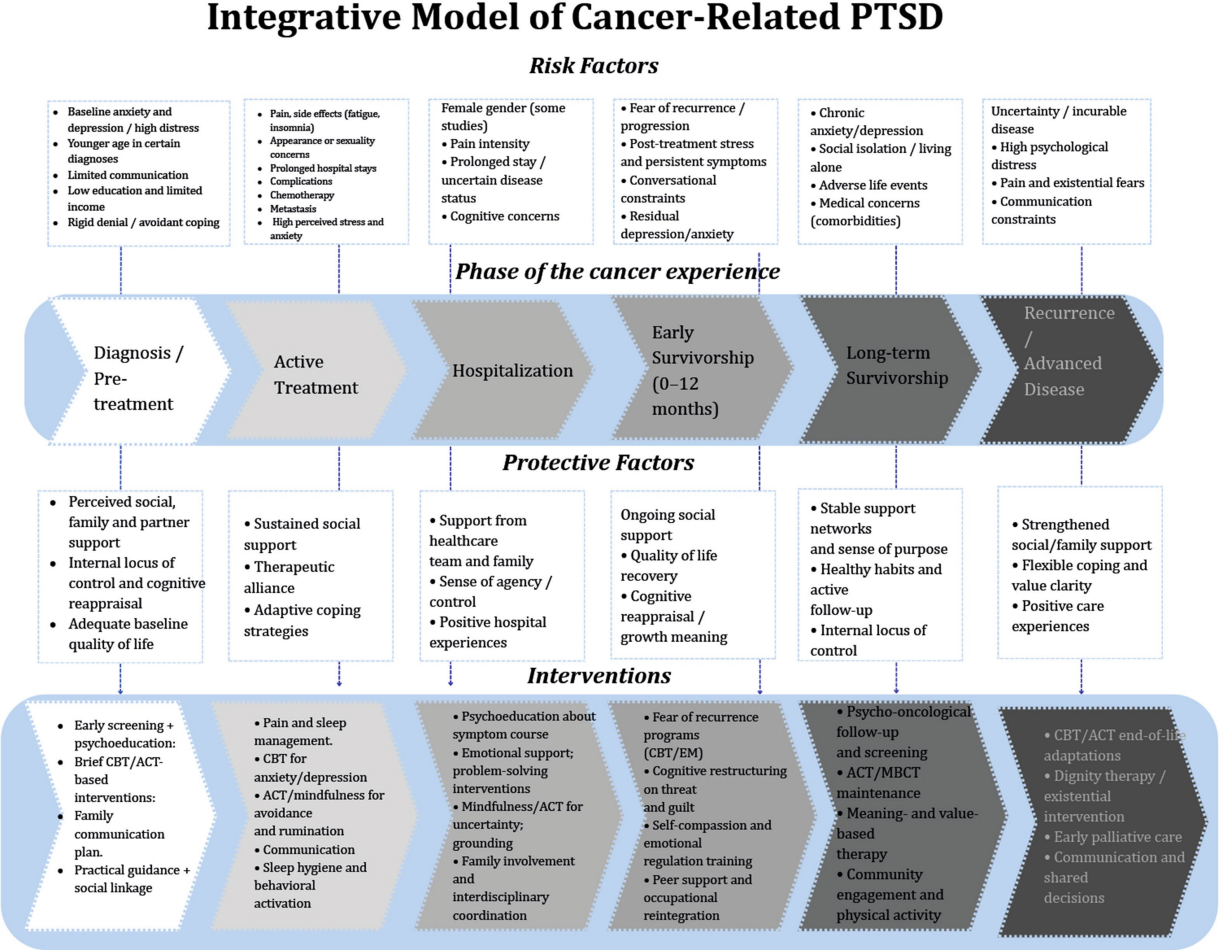
Integrative model of cancer-related PTSD across the cancer trajectory.

By analyzing the interaction of various variables across three dimensions, we identified essential elements that can guide the development of comprehensive interventions at each phase of the disease trajectory to prevent PTSD, thereby improving the overall wellbeing of patients (Figure 3).

The model is built around the different stages of the cancer journey, which include diagnosis, active treatment, hospitalization, early and long-term survivorship, and finally recurrence or advanced disease. The model shows how risk factors, protective factors, and suggested psychological interventions interact with each other in each phase.

#### Risk factors across the cancer experience

The upper section of the diagram summarizes phase-specific and cross-cutting risk factors consistently associated with PTSD symptoms. Early in the cancer trajectory, patients may present with baseline anxiety or depression, heightened distress at diagnosis, younger age, limited communication with healthcare providers, and socioeconomic vulnerabilities such as low education or limited income (Brown et al., 2020; Al Amri et al., 2024). During active treatment, symptom burden which include pain, fatigue, insomnia, appearance-related concerns, prolonged hospital stays, and chemotherapy-related effects, further intensifies emotional distress (Alkan et al., 2016; Lin et al., 2017). Hospitalization and early survivorship are marked by fear of recurrence, persistent post-treatment symptoms, conversational constraints, and residual depressive or anxiety symptoms (Chan et al., 2017; Zhou et al., 2019). In long-term survivorship, risks include chronic anxiety, social isolation, adverse life events, and comorbid medical conditions (Hahn et al., 2015; De Padova et al., 2021). Finally, during recurrence or advanced disease, uncertainty, incurable illness, existential distress, and communication barriers significantly contribute to PTSD vulnerability (Kuba et al., 2017; Moschopoulou et al., 2018). These risk factors reflect findings from the review demonstrating that younger age, female gender, affective comorbidities, maladaptive coping, social constraints, and treatment burden repeatedly predict elevated PTSD symptoms across cancer populations (Swartzman et al., 2017b; Brown et al., 2020; Jung et al., 2021).

#### Protective factors across phases

The central portion outlines protective social and psychological resources that mitigate PTSD symptoms. Early phases highlight the importance of perceived social/family support, internal locus of control, and positive baseline quality of life, consistent with evidence showing that subjective support is a robust buffer against PTSD (Swartzman et al., 2017b; Xiao et al., 2025). As patients transition into treatment and hospitalization, therapeutic alliance, adaptive coping, and support from healthcare teams contribute to sense of agency and emotional stability.

During early and long-term survivorship, quality-of-life recovery, cognitive reappraisal, stable support networks, healthy routines, and value-based living become increasingly central (El-Jawahri et al., 2016; MoshirPanahi et al., 2020). In advanced disease, strengthened family support, flexible coping strategies, and meaning-centered experiences serve as protective factors against trauma-related distress (Richardson et al., 2016; Acquati et al., 2020).

#### Interventions across the trajectory

The lower section includes evidence-based psychosocial interventions for each stage of cancer, based on what has been found to be important. Studies indicate that initial psychosocial screening, psychoeducation, brief cognitive-behavioral therapy (CBT)/acceptance and commitment therapy (ACT) interventions, family communication, and guidance can assist individuals in managing uncertainty and enhancing their coping skills (Nipp et al., 2018). Interventions during treatment concentrate on pain and sleep management, CBT/ACT and mindfulness, and behavioral activation for both physical and emotional symptoms (Reis et al., 2019). During hospitalization and the initial phase of survivorship, psychoeducation concerning symptoms, emotional support, cognitive restructuring, grounding techniques, and interdisciplinary care are crucial for mitigating fear of recurrence (Zhou et al., 2019). In long-term survivorship, maintenance cognitive-behavioral CBT/ACT, meaning-centered therapies, and community programs aid in adjustment (Malgaroli et al., 2023). During recurrence or end-of-life, guidance emphasizes ACT-based acceptance, existential therapy, and early palliative care to address existential distress, values, and communication needs (Williamson et al., 2021; Kallay et al., 2022).

## Discussion

The findings indicate that cancer-related PTSD does not stem from a singular event or specific vulnerability; instead, it results from the cumulative and ongoing interplay of psychological, socio-emotional, and medical-biological factors throughout the entire care continuum. The proposed integrative model indicates that risk evolves throughout the cancer journey, with patients potentially more adversely affected by a history of mental health problems (Hahn et al., 2015; De Padova et al., 2021), maladaptive cognitions (MoshirPanahi et al., 2020; Jung et al., 2021), high fear of recurrence (Zhou et al., 2019), or ineffective coping strategies (Simunovic and Ljubotina, 2020; De Padova et al., 2021). These vulnerabilities are frequently exacerbated by strained relationships (Chu et al., 2021), limited or ambivalent support, communication difficulties (Acquati et al., 2020), financial burdens, or structural inequalities (Zhou et al., 2019; Chu et al., 2021), as well as aggressive treatments (Alkan et al., 2016; Simunovic and Ljubotina, 2020), persistent symptoms (Lin et al., 2017; Ni et al., 2018), uncertain prognoses (Jung et al., 2021), or prolonged hospital stays (Esser et al., 2017).

It is acknowledged that substantial advancements have occurred in this domain due to increasing research; nevertheless, there remain gaps in knowledge that require enhancement for improved comprehension. For example, identifying variables that link sociodemographic factors with PTSD trajectories among various populations displaying unique cultural characteristics. Simultaneously, an expanding corpus of evidence indicates that spirituality and spiritual wellbeing, meaning-making processes, psychological resilience, and perceived social support may serve as protective resources that mitigate cancer-related distress, reduce post-traumatic stress symptoms, and promote post-traumatic growth across various oncological populations (Paredes and Pereira, 2018; Yang et al., 2023; Caldiroli et al., 2025).

There is insufficient comprehension regarding the potential impact of emerging therapies, such as immunotherapy and targeted treatments, on trauma-related distress or resilience over time. Neurobiological factors, such as tumor location and endocrine or inflammatory dysregulation, require additional investigation, especially regarding their interplay with emotional and cognitive symptoms. The impact of medical comorbidities on PTSD susceptibility, whether via physiological strain or reduced psychological resilience, necessitates additional examination.

Our model facilitates the incorporation of concise, validated screening instruments for PTSD, anxiety, depression, and fear of recurrence at critical junctures throughout the disease trajectory. Instead of a universal intensive assessment, a stepped approach is necessary: routine screening to find patients and caregivers who have more severe symptoms or are more vulnerable, followed by targeted, evidence-based interventions for those who are at higher risk or are in a lot of pain. Incorporating trauma-informed principles (such as anticipating threat cues, providing clear information, involving patients in decision-making, and so on) can mitigate iatrogenic stress and enhance adherence, communication, and quality of life.

Training for doctors, nurses, psycho-oncologists, and other health care workers should include basic information about PTSD related to cancer, high-risk profiles, caregiver distress, and how structural and cultural factors affect how symptoms show up and how people seek help. Training in empathic, honest communication, delivering bad news, shared decision-making, and recognizing trauma-related distress can improve early identification and referral. Focusing on the patient-caregiver relationship and team interactions as possible sources of safety or stress is in line with trauma-informed care and does not put too much pressure on clinicians.

Reported rates ought to be interpreted as context- and method-dependent indicators rather than exact standards, and subsequent research should integrate culturally validated instruments with locally co-developed tools and perspectives, explicitly examining cultural and linguistic moderation in multicenter, cross-cultural frameworks.

Evidence supports the incorporation of mental health into oncology standards of care at the policy level. This includes rules for stepped care and clear guidelines for when to refer someone for specialized treatment. Institutions ought to contemplate policies that tackle financial toxicity, continuity of care, and safeguarding against fragmented communication, as these elements influence susceptibility to enduring PTSD. Placing cancer-related PTSD within institutional quality and safety frameworks, instead of viewing it as a personal deficiency, can facilitate enduring resource distribution, interdisciplinary cooperation, and the assessment of care models based on the conceptual framework of this review.

Comprehending PTSD through this multifaceted perspective offers a foundation to recognize susceptible patients, predict distress trajectories, and formulate targeted interventions that address both neurobiological mechanisms and alterable psychosocial determinants. This method lays the groundwork for creating culturally sensitive, evidence-based strategies to lessen the psychological effects of cancer and improve survival rates.

### Limitations

Grounded in a biopsychosocial perspective, this review amalgamates various determinants throughout the cancer continuum, connecting risk indicators to trauma-informed care and emphasizing the caregiver context, thereby facilitating a transition from singular predictors to stratified methodologies. Nonetheless, conclusions are constrained by the inclusion of English-language studies, variability in designs, settings, instruments, and cutoffs that hindered meta-analysis and may either exaggerate or understate prevalence estimates. Evidence is concentrated on breast cancer and affluent settings, whereas minority groups, under-researched tumors, and survivorship/recurrence trajectories are relatively scarce. Self-report measures are predominant, leading to potential misclassification. Finally, there is not much mechanistic and treatment-specific data, which makes it hard to make causal claims and gives clinical recommendations less accuracy.

## Conclusion

This narrative review emphasizes the investigation of cancer-related PTSD through a comprehensive framework encompassing multiple disease dimensions. Organizing evidence across these domains elucidated the interaction between vulnerabilities and protective factors throughout the cancer trajectory, providing a comprehensive understanding of the emergence of trauma in certain patients. This multidimensional viewpoint offers a more comprehensive and clinically pertinent synthesis than singular predictors, highlighting the significance of trauma-informed methodologies in oncology.

Our findings indicate the necessity for culturally sensitive assessment instruments and research extending beyond Western and East Asian populations. Investigating cancer-related PTSD in underrepresented populations, including Latin American communities, would elucidate the influence of sociocultural norms, structural conditions, and beliefs on distress, coping mechanisms, and access to support. In addition, we recognize the importance of incorporating variables such as spirituality in future research. This element can shape coping processes during illness and foster resilience, potentially functioning as protective factors against PTSD. Despite their relevance, they were not included as key variables in the studies reviewed. Incorporating these insights into stage-specific interventions can improve prevention and detection efforts, strengthen the therapeutic alliance, and promote flexible, culturally sensitive care that enhances the quality of life for patients and families dealing with the psychological challenges of cancer.
