# Supplementary material for: Cancer as trauma: multidimensional determinants of PTSD across the disease course. A narrative integrative review
Source: Front Psychol. 2026 Jan 6;16:1719291. doi: 10.3389/fpsyg.2025.1719291 (PMC12816368; doi:10.3389/fpsyg.2025.1719291)
Supplement: Supplementary file 1 [file Supplementary_file_1.docx]

Table S1. Strategy search

| **PubMed**  Searching: 17 March 2025  No restrictions for date and language | |
| --- | --- |
| **Query** | **Related terms to identifying population** |
| #1 | (("neoplasms"[MeSH Terms] OR "cancer"[Title/Abstract] OR "tumor"[Title/Abstract] OR "malignancy"[Title/Abstract] OR "oncology"[Title/Abstract] OR "carcinoma"[Title/Abstract] OR "neoplasm*"[Title/Abstract])) |
|  | **Related terms to exposure** |
| #2 | (("stress disorders, post-traumatic"[MeSH Terms] OR "PTSD"[Title/Abstract] OR "post-traumatic stress disorder"[Title/Abstract] OR "posttraumatic stress disorder"[Title/Abstract] OR "post-traumatic stress"[Title/Abstract] OR "cancer-related PTSD"[Title/Abstract] OR "post-cancer trauma"[Title/Abstract])) |
|  | **Related terms to outcomes** |
| #3 | (("risk factors"[MeSH Terms] OR "predictors"[Title/Abstract] OR "predictive factors"[Title/Abstract] OR "prognostic factors"[Title/Abstract] OR "risk prediction"[Title/Abstract] OR "determinants"[Title/Abstract] OR "associated factors"[Title/Abstract] OR "psychosocial factors"[Title/Abstract])) |
| #4 | #1 AND #2 AND #3 |

| **Scopus**  Searching: 17 March 2025  No restrictions for date and language | |
| --- | --- |
| **Query** | **Related terms to identifying population** |
| #1 | TITLE-ABS-KEY(neoplasm* OR cancer OR tumor* OR tumour* OR malignan* OR oncology OR carcinoma*) |
|  | **Related terms to exposure** |
| #2 | TITLE-ABS-KEY(PTSD OR "post-traumatic stress disorder" OR "posttraumatic stress disorder" OR "post-traumatic stress" OR "cancer-related PTSD" OR "post-cancer trauma") |
|  | **Related terms to outcomes** |
| #3 | TITLE-ABS-KEY("risk factor*" OR predictor* OR "predictive factor*" OR "prognostic factor*" OR "risk prediction" OR determinant* OR "associated factor*" OR "psychosocial factor*") |
| #4 | #1 AND #2 AND #3 |

| **PsycINFO**  Searching: 17 March 2025  No restrictions for date and language | |
| --- | --- |
| **Query** | **Related terms to identifying population** |
| #1 | (cancer OR neoplasm* OR tumor* OR tumour* OR malignan* OR oncolog* OR carcinoma*).ti,ab. |
|  | **Related terms to exposure** |
| #2 | (PTSD OR "posttraumatic stress disorder" OR "post-traumatic stress disorder" OR "post-traumatic stress" OR (post* adj2 trauma* adj2 stress adj2 disorder*) OR "cancer-related PTSD" OR "post-cancer trauma").ti,ab. |
|  | **Related terms to outcomes** |
| #3 | ("risk factor*" OR predictor* OR "predictive factor*" OR "prognostic factor*" OR "risk prediction" OR determinant* OR "associated factor*" OR "psychosocial factor*").ti,ab. |
| #4 | #1 AND #2 AND #3 |
